# Distinguishing methicillin-resistant *Staphylococcus aureus* from methicillin-sensitive strains by combining Fe_3_O_4_ magnetic nanoparticle-based affinity mass spectrometry with a machine learning strategy

**DOI:** 10.1007/s00604-024-06342-z

**Published:** 2024-04-18

**Authors:** Wei-Hsiang Ma, Che-Chia Chang, Te-Sheng Lin, Yu-Chie Chen

**Affiliations:** 1 Department of Applied Chemistry, National Yang Ming Chiao Tung University, Hsinchu, 300 Taiwan; 2 Department of Applied Mathematics, National Yang Ming Chiao Tung University, Hsinchu, 300 Taiwan; 3 Institute of Artificial Intelligence Innovation, National Yang Ming Chiao Tung University, Hsinchu, 300 Taiwan; 4 National Center for Theoretical Sciences, National Taiwan University, Taipei, 10617 Taiwan; 5 International College of Semiconductor Technology, National Yang Ming Chiao Tung University, Hsinchu, 300 Taiwan

**Keywords:** Fe_3_O_4_ MNPs, *Staphylococcus aureus*, MRSA, MALDI-MS, Machine learning, Neural network

## Abstract

Pathogenic bacteria, including drug-resistant variants such as methicillin-resistant *Staphylococcus aureus* (MRSA), can cause severe infections in the human body. Early detection of MRSA is essential for clinical diagnosis and proper treatment, considering the distinct therapeutic strategies for methicillin-sensitive *S. aureus* (MSSA) and MRSA infections. However, the similarities between MRSA and MSSA properties present a challenge in promptly and accurately distinguishing between them. This work introduces an approach to differentiate MRSA from MSSA utilizing matrix-assisted laser desorption ionization mass spectrometry (MALDI-MS) in conjunction with a neural network-based classification model. Four distinct strains of *S. aureus* were utilized, comprising three MSSA strains and one MRSA strain. The classification accuracy of our model ranges from ~ 92 to ~ 97% for each strain. We used deep SHapley Additive exPlanations to reveal the unique feature peaks for each bacterial strain. Furthermore, Fe_3_O_4_ MNPs were used as affinity probes for sample enrichment to eliminate the overnight culture and reduce the time in sample preparation. The limit of detection of the MNP-based affinity approach toward *S. aureus* combined with our machine learning strategy was as low as ~ 8 × 10^3^ CFU mL^−1^. The feasibility of using the current approach for the identification of *S. aureus* in juice samples was also demonstrated.

**Graphical Abstract:**

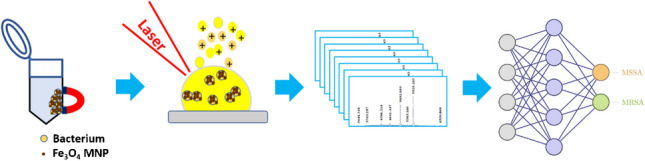

**Supplementary Information:**

The online version contains supplementary material available at 10.1007/s00604-024-06342-z.

## Introduction

*Staphylococcus aureus* has been ranked as the number one pathogen that causes the most deaths related to bacterial infections globally [1]. Generally, methicillin-resistant *S. aureus* (MRSA) strains are usually called superbugs, which have caused difficulties in treating patients infected with such pathogens [2]. Given that the antibiotics used to treat methicillin-sensitive *S. aureus* (MSSA) and MRSA are very different [3], it is vital to distinguish *S. aureus* from its drug-resistant strains. The standard methods that have been applied to test bacteria with drug-resistance are the bacterial culture-based methods—antimicrobial susceptibility testing, such as broth dilution and disk diffusion test [4]. These methods can provide the minimum inhibitory concentration (MIC) of different drugs against specific bacteria [5, 6]. However, they are time-consuming since overnight culture is required. With the development of biotechnology, polymerase chain reaction (PCR) is an alternative method that can be used to distinguish different *S. aureus* strains [7]. PCR can amplify specific DNA sequences for different strains of *S. aureus*. However, it requires specific primers for target bacteria, takes a few hours to complete the analysis, and requires professional personnel to operate the instrument. Surface plasmon resonance has also been used to distinguish *S. aureus* [8]. Nevertheless, its experimental steps are complicated, including the requirements of conducting DNA extraction and PCR. Analytical methods that can be used to rapidly distinguish MRSA from MSSA are still in high demand.

Mass spectrometry (MS) has been used to characterize bacteria [9–17]. Bacteria can be identified based on their fingerprint mass spectra in terms of their protein or metabolite profiles [9–17]. Matrix-assisted laser desorption/ionization (MALDI)-MS [9–13, 15–17] has been extensively used to detect intact bacterial cells because of its simplicity and speed. Unlike PCR, MALDI mass spectra of intact bacterial cells can be obtained in a few minutes. Protein profiles derived from bacteria shown in the MALDI fingerprint mass spectra possess excellent distinguishing capability in the bacterial species levels. However, it is still a challenge to distinguish different strains of bacteria. Thus, to explore suitable strategies that can be used to solve this challenging issue is still necessary. One possible solution is principal component analysis (PCA), which has been used to classify bacteria with good identification capabilities [18, 19]. With the increasing reliability of machine learning technology, various supervised learning strategies, including support vector machines and random forests, have also been applied to differentiate bacteria at the strain level, achieving an accuracy of around 90% [20–27]. Nevertheless, efforts are still required to improve the identification power and reduce the analysis time.

Most studies stated above were focused on the examination of the bacteria obtained after overnight culture [20–27]. It would be desirable if the time spent on overnight culture could be eliminated or reduced when analyzing real-world samples. Thus, affinity methods that can be used to selectively enrich bacteria from sample solutions by eliminating overnight cultures have been developed [13, 15, 28]. Magnetic nanoparticles (MNPs) such as functional Fe_3_O_4_ MNPs [13, 15, 28] have been extensively used as affinity probes in the enrichment of trace bacteria from sample solutions owing to their magnetism for ease of isolation of MNP-bacterium conjugates. Bare Fe_3_O_4_ MNPs also exhibit affinity toward bacteria, which are rich in oxygen-containing functional groups. The interaction between bare Fe_3_O_4_ MNPs and bacteria arises from the high affinity between Fe^3+^ on the MNPs and oxygen-containing functional groups (e.g., phosphates) on the bacterial surfaces according to the Hard Soft Acid Base theory [29]. Moreover, Fe_3_O_4_ MNPs are easy to prepare and synthesize [13, 15, 28], so they should be suitable probes for enrichment of bacteria from sample solutions for MALDI-MS analysis. In this study, we used Fe_3_O_4_ MNPs as affinity probes to enrich bacteria, followed by MALDI-MS analysis. To shorten the analysis time, microwave-heating [30–32] was used to accelerate the trapping of bacteria by the magnetic probes. The MS results were processed by using a machine-learning model. Our approach began with assembling a comprehensive dataset involving the MALDI spectra of four distinct *S. aureus* strains. We employ a neural network-based classification model to process the dataset. A distinctive feature of our data lies in its nature as a binary classification task, albeit with four labeled categories. Therefore, we employ the quaternary classification model for the training task and subsequently convert the model’s prediction results into binary classification. The established dataset was utilized to examine whether the sample containing trace bacteria can be identified using this approach. Using Fe_3_O_4_ MNP-based probes against target bacteria under microwave-heating incubation can significantly reduce the analysis time from several hours to just a couple of minutes. The current approach can overcome the time-consuming overnight culture required for the preparation of bacterial samples. Additionally, it demonstrates the possibility of employing MNP-based enrichment of trace bacteria in real-world samples for rapid distinguishing between MRSA and MSSA.

## Experimental section

### Chemicals and reagents

Ferrous (II) chloride tetrahydrate, hydrochloric acid (36.5–38.0%), tris(hydroxymethyl) aminomethane (Tris), and Tris hydrochloride were purchased from J. T. Baker (Phillipsburg, NJ, USA). Acetonitrile, ammonium hydroxide solution (30 ~ 33%), α-cyano-4-hydroxycinnamic acid (CHCA), iron (III) trichloride hexahydrate, and trifluoroacetic acid (TFA) were purchased from Merck (Darmstadt, Germany), Fluka (Charlotte, NC, USA), Sigma-Aldrich (St. Louis, MO, USA), Alfa Aesar (Massachusetts, USA), and Duksan (Ansan, South Korea), respectively. Ethanol was purchased from Echo (Miaoli, Taiwan), whereas pure water was purchased from Taisun (Changhua, Taiwan). Tryptic soy broth (TSB) was purchased from Himedia (Kennett Square, PA, USA). Yeast extract was purchased from Alpha Biosciences (Baltimore, MD, USA).* S. aureus* (an MSSA strain) and methicillin-resistant *S. aureus* (MRSA) were obtained from the Tzu-Chi Hospital (Hualien, Taiwan) and provided by Prof. P.-J. Tsai (NCKU, Taiwan). The other two MSSA strains, including *S. aureus* ATCC 6538DR (BCRC 10823) and *S. aureus* ATCC 12692 (BCRC 10831), were purchased from the Bioresource Collection Research Center (BCRC) (Hsinchu, Taiwan). Apple juice was purchased from a local shop.

### Instrumentation

All the MALDI mass spectra were acquired from an Autoflex III MALDI-time of flight (TOF) mass spectrometer (Bruker Daltonics, Bremen, Germany). The mass spectrometer was equipped with a Nd:YAG laser with a wavelength of 355 nm. All the samples were conducted by the linear TOF with the positive ion mode. The voltages on the mass spectrometer were set as follows: ion source 1, 20.00 kV; ion source 2, 18.65 kV; lens, 6.80 kV. The laser frequency was set at 100 Hz. Bacteria were cultured in an incubator (Deng Yng DB60, Taipei, Taiwan) at 37 ℃. The optical density of bacterial suspension at the wavelength of 600 nm (OD_600_) was recorded using either a Biochrom WPA CO8000 (Cambridge, UK) or a Biosan DEN-600 Photometer (Riga, Latvia).

### Bacterial culture

*S. aureus* (clinical, BCRC 10823, BCRC 10831, and MRSA clinical strains) were cultured on the agar plate containing TSB and yeast extract (TSBY) at 37℃ for ~ 20 h. TSBY agar plates were prepared by dissolving TSB (10 g), yeast extract (2 g), and agar (10 g) in deionized water (400 mL), followed by sterilization and pulling to individual Petri dishes. Freshly harvested bacteria were used for the experiments in the study.

### MALDI-MS analysis of model bacteria

A couple of *S. aureus* colonies prepared above were mixed with TFA (3%, 20 µL). The resulting bacterial sample (1.5 µL) was mixed with the MALDI matrix (1.5 µL), i.e., CHCA (25 mg mL^−1^), which was prepared in acetonitrile/3% TFA (2:1, v/v). The resulting mixture (1.5 µL) was deposited on the MALDI plate. After solvent evaporation, the sample was ready for MALDI-MS analysis, in which the linear TOF was operated with the positive ion mode. The laser frequency was set at 100 Hz. Each mass spectrum acquired with the *m/z* range of 3000–8000 was collected from 3000 to 10,000 laser shots until the peak at *m/z* 6888 reaching the intensity of ~ 2000. 63, 52, 54, and 64 mass spectra derived from *S. aureus* clinical strain, *S. aureus* BCRC 10823, *S. aureus* BCRC 10831, and MRSA clinical strain, respectively, were acquired to establish the machine learning dataset.

### Using a machine learning strategy to build a classification model for four model *S. aureus* strains

The classification model and deep SHAP analysis were implemented using the Python programming language. The machine learning model comprises three parts. The initial stage involves data preprocessing, followed by utilizing a neural network quaternary classification model in the second part. Lastly, the third component involves a post-processing binary classification model. The first and last parts are pre-defined and do not require training, while the classification model of the second part contains unknown parameters that need to be learned from the data. The details of the machine learning algorithm used to classify the four *S. aureus* based on our MALDI-MS data were provided in the Appendix I in Electronic Supporting Material (ESM).

### Using Fe_3_O_4_ MNPs as affinity probes to trap model bacteria

The freshly harvested bacteria, as prepared above, were centrifuged at 6000 rpm for 3 min. The bacteria cells were rinsed with Tris buffer (5 mL, pH 6) and centrifuged at 6000 rpm for 3 min, repeating the process for two cycles. After the rinse, the bacterial cells were suspended in Tris buffer with an OD_600_ equal to 1. The bacterial suspension was further diluted to the desired concentrations before the experiments. Fe_3_O_4_ MNPs were generated based on the protocol reported previously [33]. The details of the preparation of Fe_3_O_4_ MNPs were provided in the Appendix II in ESM. When using Fe_3_O_4_ MNPs as affinity probes against the model bacteria, Fe_3_O_4_ MNPs (~ 50 µg) were added to the sample (1 mL) containing *S. aureus*. The mixture was vortex-mixed for a few seconds and subjected to microwave-heating (power, 180 W) for 2 min. The resulting MNP-bacterium conjugates were magnetically isolated using an external magnet for approximately 5 min. The supernatant was then removed, TSBY (1 mL) was added, and the solution was incubated for another 4–6 h. After incubation, the remaining MNPs were discarded by magnetic isolation, whereas the remaining supernatant containing newly grown bacteria was centrifuged at 6000 rpm for 5 min, followed by rinse with Tris buffer (20 mM, pH 6) for four cycles. After rinsing, a new batch of Fe_3_O_4_ MNP (~ 30 µg) and Tris buffer (20 mM, ~ 0.9 mL) were added to the rinsed bacterial cells to have a final volume of 1 mL. The mixture was vortex-mixed for a few seconds, followed by microwave-heating. The MNP-bacterium conjugates were magnetically isolated by placing an external magnet for 5 min to remove the supernatant, followed by centrifugation at 6000 rpm for 5 min, and the supernatant was discarded. The resulting MNP-bacterium conjugates were mixed with the MALDI matrix (1.5 µL). The MALDI matrix was prepared by dissolving CHCA (25 mg mL^−1^) in acetonitrile/3% TFA (2:1, v/v). The resulting mixture was deposited on the well on the MALDI plate. After solvent evaporation, the sample was ready for MALDI-MS analysis.

### Analysis of simulated real samples

Apple juice was used to prepare simulated real samples. That is, 100-fold diluted apple juice samples prepared in Tris buffer (20 mM, pH 6) were spiked with model bacteria with different concentrations. The experimental steps using Fe_3_O_4_ MNPs as affinity probes followed by MALDI-MS analysis were similar to those steps stated above. The resulting mass spectral data were input to the established database using the developed machine-learning strategy.

## Results and discussion

### MALDI mass spectra of four model *S. aureus*

**Fig. 1:**
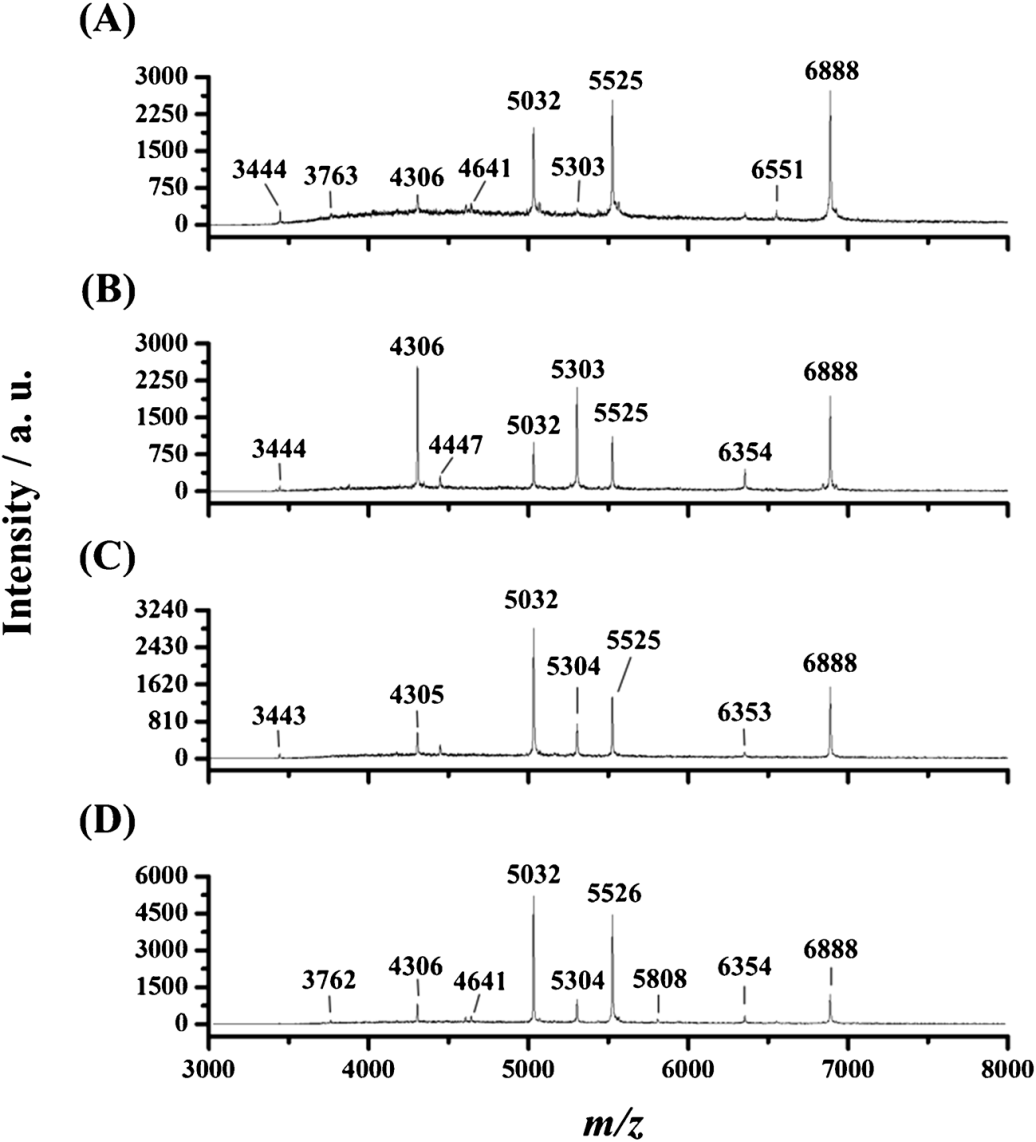
Representative MALDI mass spectra of *S. aureus* **A** clinical (*n* = 63), **B** BCRC 10823 (*n* = 52), **C** BCRC 10831 (*n* = 54), and **D** MRSA clinical strains (*n* = 64). “*n*” stands for the number of the mass spectra of the individual bacterial strains that were acquired. One or two *S. aureus* colonies were mixed with 3% TFA (20 µL). The resulting bacterial sample (1.5 µL) was then mixed with the MALDI matrix CHCA (25 mg mL^−1^), prepared in acetonitrile/3% TFA (2:1, v/v). This mixture (1.5 µL) was deposited onto the MALDI plate. After solvent evaporation, the sample was ready for MALDI-MS analysis using the linear TOF in positive ion mode

Four *S. aureus* strains, including one clinical strain, BCRC 10823, BCRC 10831, and one MRSA strain, were selected as the model bacteria in this study. The MIC of *S. aureus* against oxacillin has been used as the guideline to distinguish MSSA from MRSA [6]. The MICs of *S. aureus* clinical strain, BCRC 10823, BCRC 10831, and MRSA clinical strain toward oxacillin were 0.25, 0.25, 0.25, and 16 μg mL^−1^, respectively (ESM Figure S1), whereas they were < 0.125, 0.5, 0.5, and 2 μg mL^−1^, respectively, toward vancomycin (ESM Figure S2). That is, these four strains are vancomycin sensitive, whereas *S. aureus* clinical strain, BCRC 10823, and BCRC 10831 were confirmed as MSSA. Figure 1 shows the representative MALDI spectra of these four *S. aureus* strains obtained from the linear MALDI-TOF operated at the positive ion mode. The peaks at *m/z* 5032, 5525, and 6888 were observed in all the mass spectra of these four *S. aureus* strains. Given that the peak at *m/z* 6888 was the major peak among these bacterial strains, we acquired the MALDI mass spectra of individual samples with intensities of this peak up to ~ 2000 during MALDI-MS analysis. It should be noted that the mass resolution used in the linear mode was less than 2000. Consequently, the *m/z* values derived from the same peak may have a discrepancy of 1–3 amu. For example, the peak at *m/z* 5526 in Fig. 1D should be the same as the peak at *m/z* 5525 shown in Fig. 1A–C. The peak at *m/z* 4305 in Fig. 1C should be the same at *m/z* 4306 shown in Fig. 1A, B, and D. There was not a significant difference in mass spectral profiles among MSSA and MRSA strains. However, the relative intensity of ion peaks in the mass spectra varied among the strains. Therefore, we further employed a machine-learning strategy to investigate whether these different *S. aureus* strains could be distinguished from each other.

### Neural network-based classification model for *S. aureus* strains

**Scheme 1:**
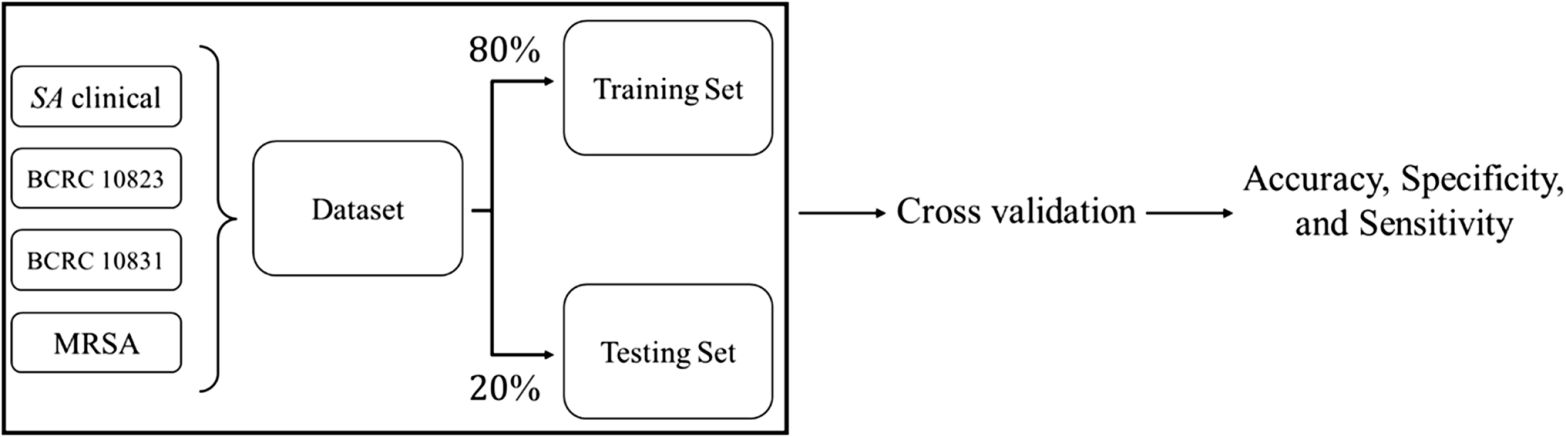
Schematic illustration of random data separating and testing process (*SA* stands for *S. aureus*)

The total number of the MALDI spectra derived from those four *S. aureus* strains was 233. Scheme 1 shows the schematic illustration to describe how the data was selected and processed in the classification model. We used cross-validation to check the accuracy of the model.

The model was trained 20 times. At each time, the data was divided into training and test sets. We randomly selected 80% of the MALDI spectra as the training dataset, while the remaining 20% constituted the test dataset. A classification model was trained using training data, whereas accuracy, sensitivity, and specificity were calculated using test data. The final performance metrics are averaged over the 20 iterations.

Table 1 indicates that the accuracy for identifying four *S. aureus* strains, including clinical strains BCRC 10823 and BCRC 10831, as well as the MRSA clinical strain, was 96.92%, 97.27%, 92.27%, and 96.54%, respectively. In Table 2, MSSA and MRSA were classified by placing *S. aureus* clinical strains BCRC 10823 and BCRC 10831 in the MSSA group. Table 3 presents the resulting accuracy, sensitivity, and specificity as 97.92%, 98.43%, and 96.54%, respectively, based on the outcomes listed in Table 2. These findings demonstrate that the model effectively predicts the target strains using the established dataset. Furthermore, the machine-learning strategy achieved high accuracy, sensitivity, and specificity in classifying MSSA and MRSA.

**Table 1:** Model confusion matrix of testing set for four model *S. aureus* strains. SA stands for* S. aureus*

	Clinical SA Predicted	SA 10823 Predicted	SA 10831 Predicted	MRSA Predicted	Accuracy (%)
Clinical SA	252	0	0	8	96.92
SA 10823	5	214	1	0	97.27
SA10831	7	7	203	3	92.27
MRSA	9	0	0	251	96.54

**Table 2:** Classification results for MSSA and MRSA

	MSSA predicted	MRSA predicted
MSSA	689 (a)	11 (b)
MRSA	9 (c)	251 (d)

Accuracy= ((a+d)/(a+b+c+d))%; Sensitivity= (a/(a+b))%; Specificity= (d/(c+d))%

**Table 3:** Accuracy, sensitivity, and specificity in classifying MSSA and MRSA

	Accuracy	Sensitivity	Specificity
Test results	97.92%	98.43%	96.54%

Accuracy = ((a + d)/(a + b + c + d))%; Sensitivity = (a/(a + b))%; Specificity = (d/(c + d))%

### Characterization of the feature peaks

**Fig. 2:**
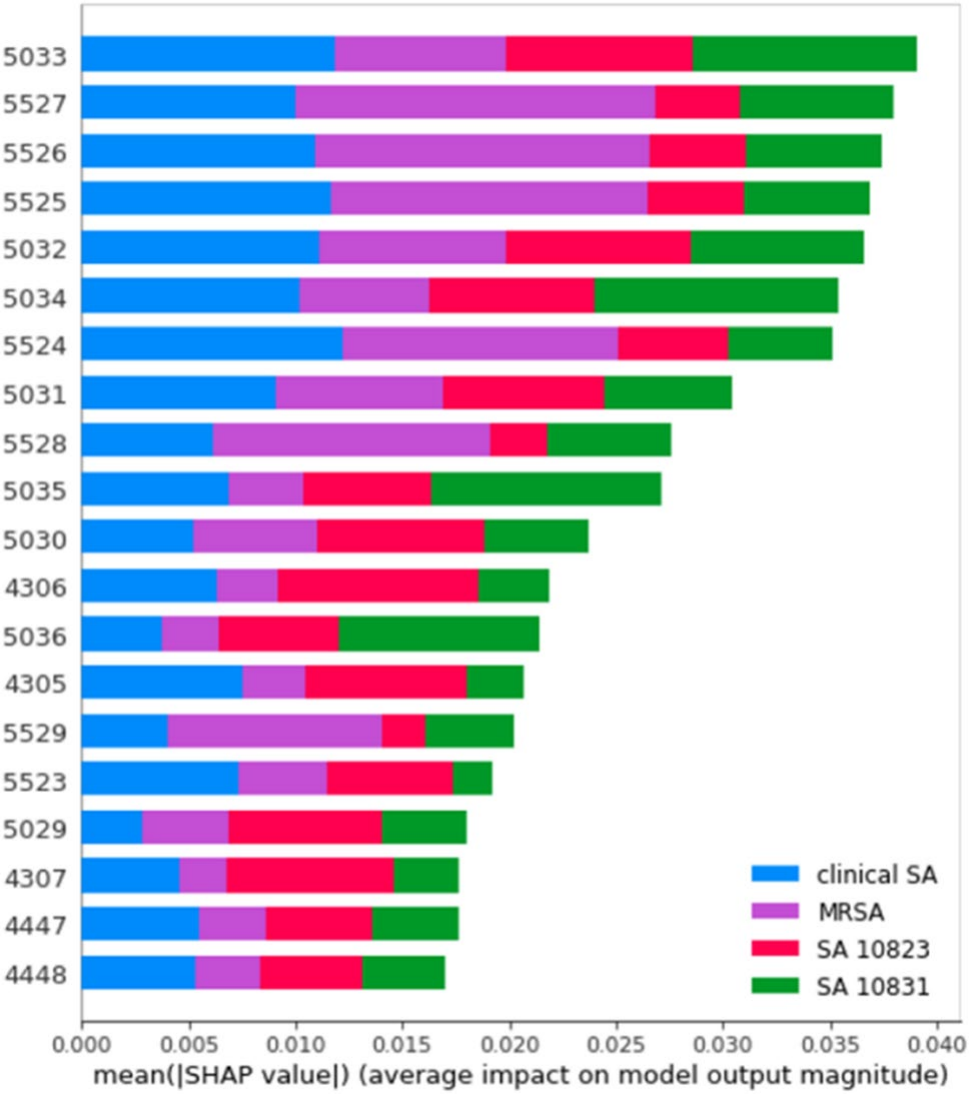
Mean absolute SHAP values in Deep SHAP results obtained from the MALDI mass spectra of four model bacterial strains. The sample preparation steps were stated in the legend shown in Fig. 1

**Fig. 3:**
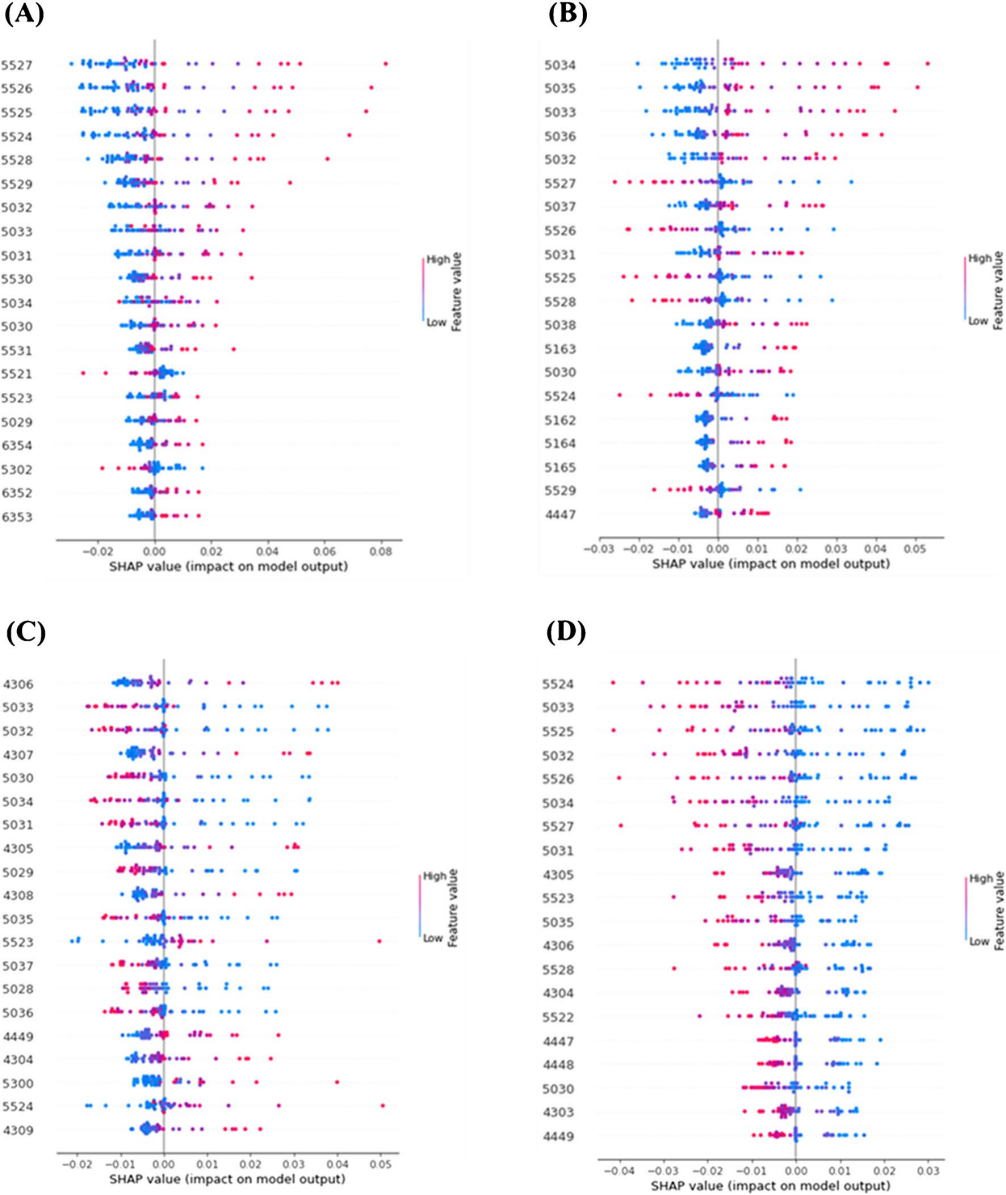
SHAP values that impact on model output for the direct MALDI mass spectra obtained from the samples containing **A** MRSA clinical strain (*n* = 64), **B** *S. aureus* BCRC 10831 (*n* = 54), **C** *S. aureus* BCRC 10823 (*n* = 52), and **D** *S. aureus* clinical strain (*n* = 63). The sample preparation steps were stated in the legend shown in Fig. 1

To determine the feature peaks in each strain, we used Deep SHapley Additive exPlanations (SHAP) to characterize the important peaks in the mass spectrum of each strain. Figure 2 shows the essential features found by Deep SHAP and the influence of these feature peaks on each strain. Figure 3 shows the model SHAP value impact on model output for each strain. The top five feature peaks in each strain are listed below: the feature peaks for clinical strain were *m/z* 5524, 5033, 5525, 5032, and 5526; for BCRC 10823 were *m/z* 4306, 5033, 5032, 4307, and 5030; for BCRC 10831 were *m/z* 5034, 5035, 5033, 5036, and 5032; and for MRSA strain were *m/z* 5527, 5526, 5525, 5524, and 5528. Let us take Figure 3A as an example; the figure classified the data into two groups: one is the target group, i.e., MRSA, listed on the positive coordinates; the other one is the non-target group (clinical, BCRC 10823 and BCRC 10831) listed on the negative coordinates. The red spots denoted the feature having a significant influence in this group, whereas the blue spots denoted the feature having a low influence. The top five features in Fig. 3A were *m/z* 5527, 5526, 5525, 5524, and 5528, derived from MRSA, which were marked with red spots at the positive coordinates. Given that we operated the MALDI mass spectrometer at the linear mode when analyzing these model bacteria, as mentioned earlier, the mass resolution was not good, resulting in the broad peak observed in Fig. 1. Thus, these discovered feature peaks were, in fact, derived from the same identity. That is, the feature peak at *m/z* 5525 standing for MRSA. The feature peak of *S. aureus* BCRC 10831, as shown in Fig. 3B, was *m/z* 5033 (*m/z* 5033 ± 2). In Fig. 3C for *S. aureus* BCRC 10823, two feature peaks were apparent at *m/z* 4306 (i.e., *m/z* 4306 and 4307) and *m/z* 5033 (i.e., *m/z* 5033, 5032, and 5030). Notably, the presence of red color spots at *m/z* 4306 and blue color spots at *m/z* 5033 in BCRC 10823 implies a more pronounced influence of the feature peaks at *m/z* 4306 and a comparatively lesser influence of those at *m/z* 5033. Examining the *S. aureus* clinical strain depicted in Fig. 3D, Deep SHAP analysis revealed multiple characteristic peaks. However, a distinctive pattern emerges: all of these features exhibit blue color spots on positive coordinates. This outcome signifies that the machine learning model initially identifies spectra resembling MRSA, BCRC 10831, and BCRC 10823. That is, those discovered ions have a low influence in identifying the *S. aureus* clinical strain. Should the spectra fail to align with these three strains, the model subsequently classifies them as belonging to the *S. aureus* clinical strain. These feature peaks enabled the classification model to distinguish different model strains.

### Using Fe_3_O_4_ MNPs as affinity probes to trap model bacteria

We further examined the possibility of using Fe_3_O_4_ MNPs as affinity probes to enrich target bacteria in the sample solution. Therefore, the time for overnight culture could be eliminated. The details of the experimental steps have been provided in the “Experimental section.” ESM Table S1 shows the binding capacity of Fe_3_O_4_ MNPs against *S. aureus* clinical strain at different pH values. The result shows that with the increase of the pH value, the binding capacity of the MNPs toward *S. aureus* was reduced. The optimal binding capacity appeared at pH 5 and 6.

**Fig. 4:**
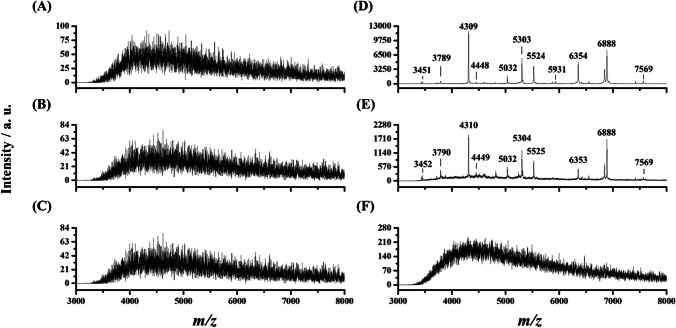
Direct MALDI mass spectra of MRSA clinical strain with the concentrations in terms of OD values of **A** 10^−1^, **B** 10^−2^, and **C** 10^−3^. MALDI mass spectra of the same bacterial samples (1 mL) with the concentrations of OD values of **D** 10^−1^, **E** 10^−2^, and **F** 10^−3^ obtained after using Fe_3_O_4_ MNPs (50 μg) to enrich trace target bacteria from the samples followed by MALDI-MS analysis. The samples were incubated under microwave heating for the MNP enrichment

Figure 4A, B, and C show the MALDI mass spectra of MRSA clinical strain with the concentrations of OD values of 10^−1^, 10^−2^, and 10^−3^, respectively, from direct MS analysis. It was apparent that no peaks were found in the mass spectra. After MNP enrichment from the sample (1 mL) containing MRSA clinical strain with the concentrations of OD values of 10^−1^ and 10^−2^, the peaks representing the target bacteria could be observed in the mass spectra (Fig. 4D and E). However, it was impossible to see any peaks when the concentration of the target bacteria was lowered to OD of 10^−3^ (Fig. 4F).

**Fig. 5:**
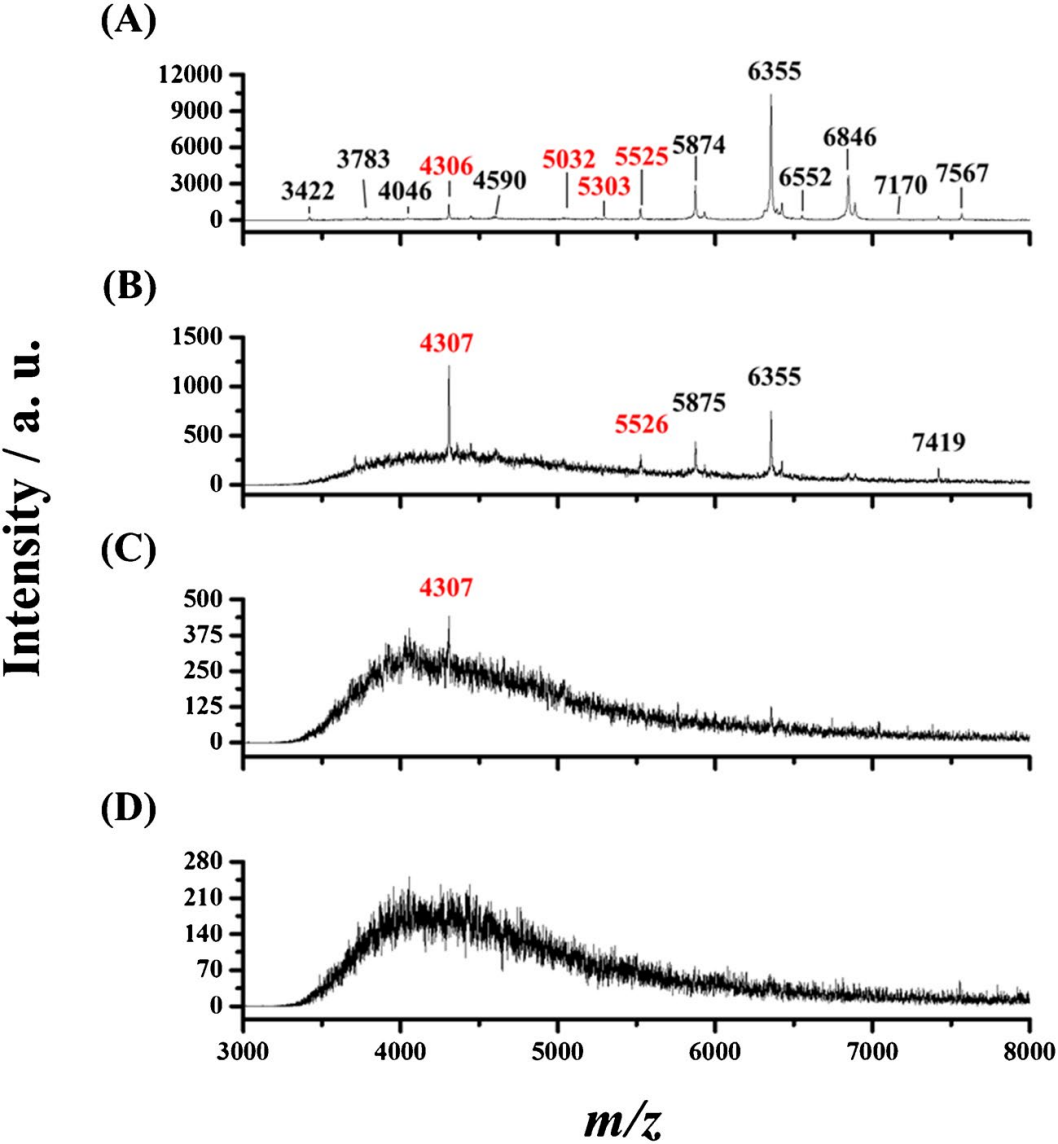
MALDI mass spectra of the samples (1 mL) containing MRSA clinical strain with the concentrations in terms of the OD values of **A** 10^−4^, **B** 10^−5^, **C** 10^−6^, and **D** 10^−7^ obtained after enriched by Fe_3_O_4_ MNPs (50 µg) under microwave-heating followed by 6-h culture

To further improve the lowest detectable concentration, we cultured the bacteria trapped on the MNPs for 6 h before MALDI-MS analysis. The detailed experiment steps were described in the “Experimental section.” MRSA clinical strain was used as the model bacteria. Figure 5 shows the resultant mass spectra of the sample containing MRSA clinical strain with different concentrations in terms of OD values of 10^−7^–10^−4^ obtained after using Fe_3_O_4_ MNPs as affinity probes for enrichment, followed by 6-h culture. Those peaks marked with red *m/z* values were derived from MRSA. Apparently, the peaks representing the MRSA appeared in the mass spectra as the concentration of the target bacterium was reduced to the OD of 10^−5^. As the concentration of the target bacterium was reduced to 10^−6^, only one peak at *m/z* 4307 derived from the MRSA clinical strain was observed in the mass spectrum. ESM Table S2 shows the classification result. When the samples containing MRSA have concentrations in terms of OD values of 10^−4^ and 10^−5^, the established model can correctly identify the target bacteria, i.e., MRSA. However, the model failed to identify the target bacteria as the bacterial concentration was further reduced to the OD value of 10^−6^. It was because many feature peaks appeared in the resulting mass spectra of the samples containing the target bacteria with a high concentration, whereas few peaks were observed in the resulting mass spectra of the samples containing a low concentration of target bacteria. These results indicated that the lowest detectable concentration of MRSA clinical strain could be reduced to the concentration with the OD value of ~ 10^−5^ (~ 4 x 10^4^ CFU mL^−1^) after enrichment, followed by a 6-h culture. The detection limit of the approach was estimated to be approximately ~8 × 10^3^ CFU mL^−1^ (OD of 1 =  ~ 4 × 10^9^ CFU mL^−1^) [34] by considering the peak at *m/z* 4307 (a signal -to-noise ratio (S/N) of 15), representing MRSA (Fig. 5B, the concentration of MRSA = OD of ~ 10^−5^) based on an S/N of 3.

### Real sample analysis

**Fig. 6:**
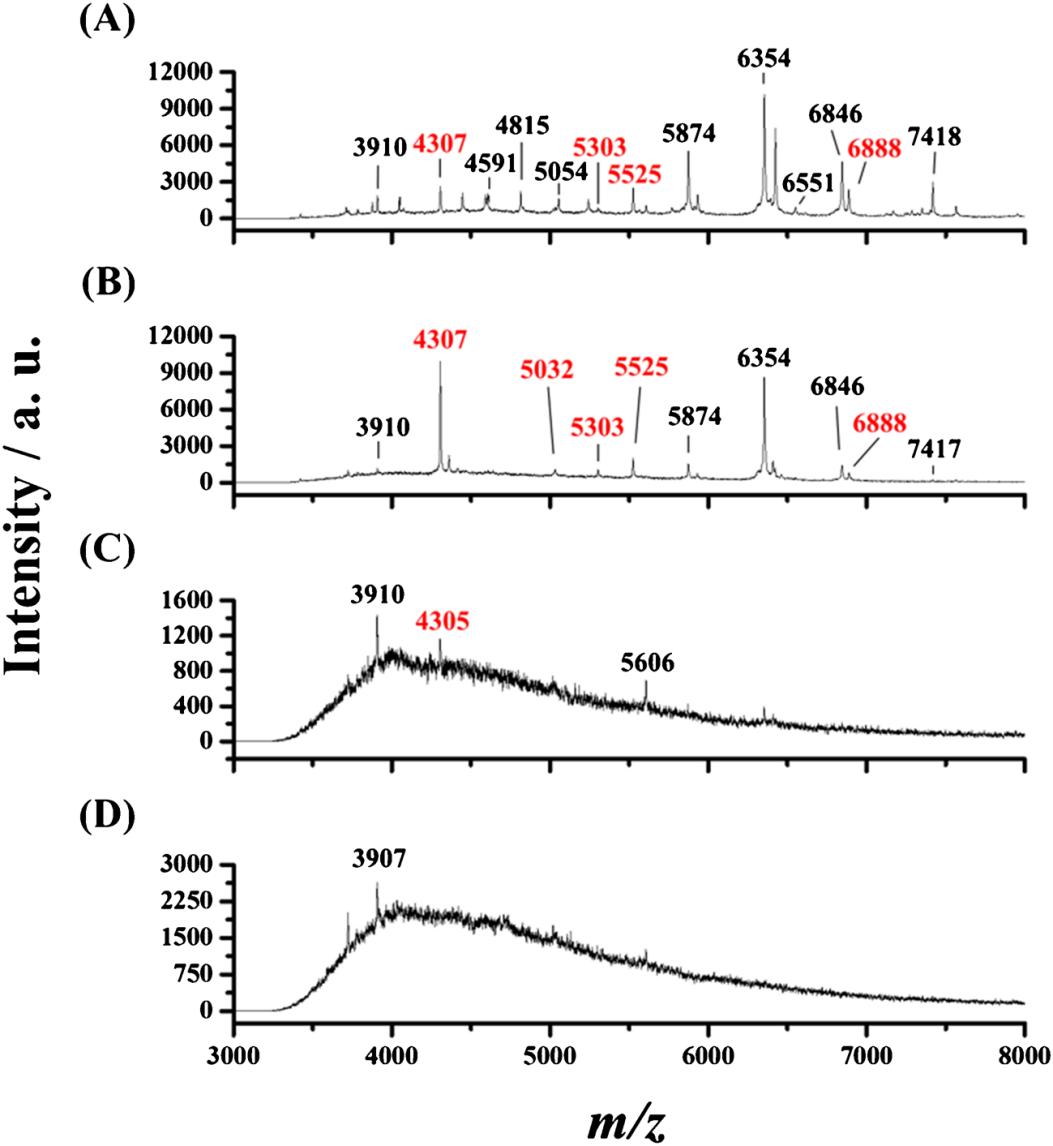
MALDI mass spectra of the 100-fold diluted juice samples (1 mL) containing MRSA clinical strain with the concentrations in terms of the OD values of **A** 10^−4^, **B** 10^−5^, **C** 10^−6^, and **D** 10^−7^ obtained after enriched by Fe_3_O_4_ MNPs (50 µg) under microwave-heating followed by 6-h culture. The peaks marked with red text were derived from MRSA

We then examined the feasibility of using the developed method to characterize target bacteria in the simulated real sample. A 100-fold diluted apple juice by Tris buffer (pH 6) was spiked with MRSA clinical strain with different concentrations and treated by our developed method, the same as that used to obtain Fig. 5. Figure 6 shows the resulting MALDI mass spectra. Many feature peaks, including *m/z* 4307, 5032, 5303, 5524, 5525, and 6888 (marked in red) that were discovered by DEEP SHAP, were observed (Fig. 3). The data obtained in Fig. 6 were processed using the established dataset based on the developed machine learning model. ESM Table S3 shows the machine learning results for identifying target bacteria using the established machine learning model. The identity of the target bacteria could be correctly identified even though the concentration of MRSA clinical strain was reduced to the OD value of 10^−5^. The results indicated the potential of using the developed method to analyze target bacteria in complex samples.

### Comparison of our approach with the existing methods

ESM Table S4 shows a list comparing the existing methods [21, 23–26, 35–37] with our approach. Although our method required a 6-h incubation time after MNP enrichment, all the existing methods [21, 23–26, 35–37] required 12–24 h before MALDI-MS analysis could be carried out. That is, MNP enrichment can be used to effectively reduce the entire time for sample preparation. Nevertheless, the current method still requires a 6-h culture for the bacterial samples with their OD <  ~ 10^−2^ (= ~ 10^7^ CFU mL^−1^)) (*cf.* Figure 4 and Fig. 5), to obtain sufficient bacterial cells for MALDI-MS analysis and correct identifications by our machine learning strategy. Thus, further efforts should be devoted to the reduction of the sample preparation time. Moreover, our method has a relatively low LOD, i.e. ~ 8 × 10^3^ CFU mL^−1^ compared with the existing methods [21, 23–26, 35–37]. The accuracy of our method was 92–97%, which was relatively good, compared with most of the existing methods [21, 23–26, 35, 36].

## Conclusions

Machine-learning strategies have been used to effectively distinguish different bacteria based on the MALDI mass spectra of intact bacterial cells. Nevertheless, time-consuming overnight culture is usually required prior to MS analysis. In this study, we have developed a method that combines affinity based-MS with a machine-learning strategy to distinguish MRSA from MSSA. Fe_3_O_4_ MNPs were demonstrated to be useful affinity probes that could be used to effectively enrich trace bacteria from the sample solution within 2 min under microwave-heating. Therefore, overnight culture time could be further reduced to 6 h for correctly identifying trace bacteria from the sample solution. Our machine-learning model demonstrated commendable classification prowess, yielding high accuracy levels for each *S. aureus* strain. We found distinctive feature peaks associated with each strain using the Deep SHAP methodology. Based on our results, the developed Fe_3_O_4_ MNP-based affinity MALDI-MS combined with a deep learning strategy provides a new method to effectively reduce the entire analysis time, which is the main advantage over the existing methods. Enrichment of target bacteria with the concentration ≧ ~ 10^7^ CFU mL^−1^ followed by MALDI-MS analysis can be completed within 10 min. However, the current method still requires 6-h culture for the bacteria samples with the concentration lower than 10^7^ CFU mL^−1^ to obtain enough cells for MALDI-MS analysis and accurate identifications with our machine learning strategy. Thus, efforts are still needed to further reduce the time in the sample preparation. Therefore, it will be possible for on-site detection of pathogenic bacteria.

### Supplementary Information

Below is the link to the electronic supplementary material.

Supplementary file1 (PDF 744 KB)
