# Supplementary material for: Distinguishing methicillin-resistant Staphylococcus aureus from methicillin-sensitive strains by combining Fe3O4 magnetic nanoparticle-based affinity mass spectrometry with a machine learning strategy
Source: Mikrochim Acta. 2024 Apr 18;191(5):273. doi: 10.1007/s00604-024-06342-z (PMC11026280; doi:10.1007/s00604-024-06342-z)
Supplement: Supplementary file 1 — Supplementary file1 (PDF 744 KB) [file 604_2024_6342_MOESM1_ESM.pdf]

Electronic Supporting Material

Distinguishing Methicillin-resistant *Staphylococcus aureus* from Methicillin-sensitive Strains by Combining Fe_3O_4 Magnetic Nanoparticle-based Affinity Mass Spectrometry with A Machine Learning Strategy

Wei-Hsiang Ma,[1#] Che-Chia Chang,[2,3#] Te-Sheng Lin,[2,4*], Yu-Chie Chen[1,5*]

[1]Department of Applied Chemistry, National Yang Ming Chiao Tung University, Hsinchu 300, Taiwan

[2]Department of Applied Mathematics, National Yang Ming Chiao Tung University, Hsinchu 300, Taiwan

[3]Institute of Artificial Intelligence Innovation, National Yang Ming Chiao Tung University, Hsinchu 300, Taiwan

[4]National Center for Theoretical Sciences, National Taiwan University, Taipei 10617, Taiwan

[5]International College of Semiconductor Technology, National Yang Ming Chiao Tung University, Hsinchu 300, Taiwan

[#]The authors contributed equally to this work.

[*]Corresponding authors

T.-S. Lin

E-mail: teshenglin@nycu.edu.tw

Tel: +886-3-5712121 ext: 56422

Y.-C. Chen

E-mail: yuchie@nycu.edu.tw

Tel: +886-3-5131527

Fax: +886-3-5723764

Appendix I

1. Appendix – Neural network-based classification model

1.1. Data preprocessing

In the first stage, we aimed to normalize the mass spectrum intensity data for each *S. aureus* and extract representative values. The intensity of each data was normalized to one, and a max-pooling process was performed to obtain integer intensity between 3000 and 8000.

1.2. Multiclass classification model

We used a feedforward fully-connected neural network as the model. The dimension of the input layer is 5001, which is consistent with the number of input intensities. The output layer has four units to implement quaternary classification. At the last layer, the results are passed into a softmax function to obtain positive values with sum one, and the output class is the one with the most significant magnitude.

For model training, we utilize the conventional cross-entropy loss function and employ the Adam optimizer.

1.3. Binary classification model

To obtain a binary classification model, we added a layer to the output of the trained quaternary classification model. This layer does not require training, and its purpose is simply to condense quaternary classification into binary classification.

1.4. Validation

We used cross-validation to verify the robustness of the model. In each experiment, we randomly selected 80% of the data as the training set and the remaining 20% as the test set. The final accuracy of our model is the average of 20 experiments.

1.5. Feature importance

We used Deep SHAP [Lundberg, Scott M and Lee, Su-In, A Unified Approach to Interpreting Model Predictions, NIPS (2017), pp.4765–4774] to analyze the model and to find the important features. In the deep SHAP analysis, the mean absolute SHAP value illustrates the importance of each feature, and the sign of the SHAP value signifies which class the feature is most crucial for.

Appendix II

Additional Experimental Details

Synthesis of Fe_3O_4 MNPs

Iron (II) chloride (0.49 g) and iron (III) chloride (0.67 g) were initially dissolved in deionized water (25 mL) in a double-necked flask. The air in the double-necked flask was pumped out, followed by putting a balloon filled with nitrogen gas on one neck. Aqueous ammonia (33%, 25 mL) was slowly injected into the flask from the other sealed neck using a syringe connected to a syringe pump with a flow rate of 0.4 mL min^{-1}. The solution in the flask was continuously stirred at room temperature for 2 h, with all processes carried out under nitrogen protection. The generated Fe_3O_4 MNPs were aggregated on the wall of the vial using an external magnet (~4000 Gauss). The supernatant was then removed. The resulting Fe_3O_4 MNPs were washed with deionized water (20 mL × 1) and ethanol (20 mL × 3). Subsequently, the MNPs were suspended in ethanol (40 mL) and stored in a refrigerator at 4 °C until use.

Table S1. The binding capacity of Fe_3O_4 MNPs toward the *S. aureus* at different pH values.

pH value	5	6	7	8	9
Binding capacity (CFU mg^{-1})	$\sim 1.38\times 10^{10}$	$\sim 1.38 \times 10^{10}$	$\sim 1.2 \times 10^{10}$	$\sim 1.08 \times 10^{10}$	$\sim 7.2 \times 10^{9}$

Table S2. Classification of the target bacteria using our machine learning strategy. "O" denotes that data was hit on the right one, whereas "X" indicates that the results were incorrect.

Samples	Results
MRSA (OD 10^{-4})	O
MRSA (OD 10^{-5})	O
MRSA (OD 10^{-6})	X
MRSA (OD 10^{-7})	X

Table S3. Identification of the target bacteria from the simulated real sample using our machine learning strategy. "O" denotes that the data was hit on the right one, whereas "X" indicated that the results were incorrect.

Samples	Results
MRSA (OD 10^{-4})	O
MRSA (OD 10^{-5})	O
MRSA (OD 10^{-6})	X
MRSA (OD 10^{-7})	X

Table S4. List of comparisons between the current work and the existing studies.

Reference number	Published Year	Target bacteria	Bacterial culture time	LODs	Number of mass spectra used in machine learning	Machine learning strategies	Accuracy
1	2002	35 different strains bacteria	24 h	-	212	Hybrid neural network	79% - 89%
2	2013	*Shigella species*/*E. coli*	18-24 h	-	138	Genetic algorithm	90% - 96%
3	2019	MRSA/MSSA	12 h	~10^4 cells μL^{-1} (= ~10^7 CFU mL^{-1})	140	BinDA, random forest	63% - 93%
4	2020	MRSA/MSSA	18-24 h	-	4858	Decision tree, random forest, Knearest Neighbor, and support vector machine	69.8% - 76.64%
5	2021	MRSA/MSSA	*-	-	548	Decision tree, polynomial regression, random forest, support vector machine	75% - 87%
6	2021	MRSA/MSSA	24 h	-	452	Support vector machine, random forest	82% - 86%
7	2022	MRSA/MSSA	24 h	-	33975	Deep neural network	97.66 ± 0.26%
8	2022	MRSA/MSSA	18-24 h	-	20359	LightGBM	**with AUC 0.78-0.88
This work	2023	MRSA/MSSA	6 h (after MNP capture)	~8 ×10^3 CFU mL^{-1}	233	Neural network	92% - 97%

* Not specified.
** This paper provides AUC, true positive rate, and true negative rate, instead of accuracy.

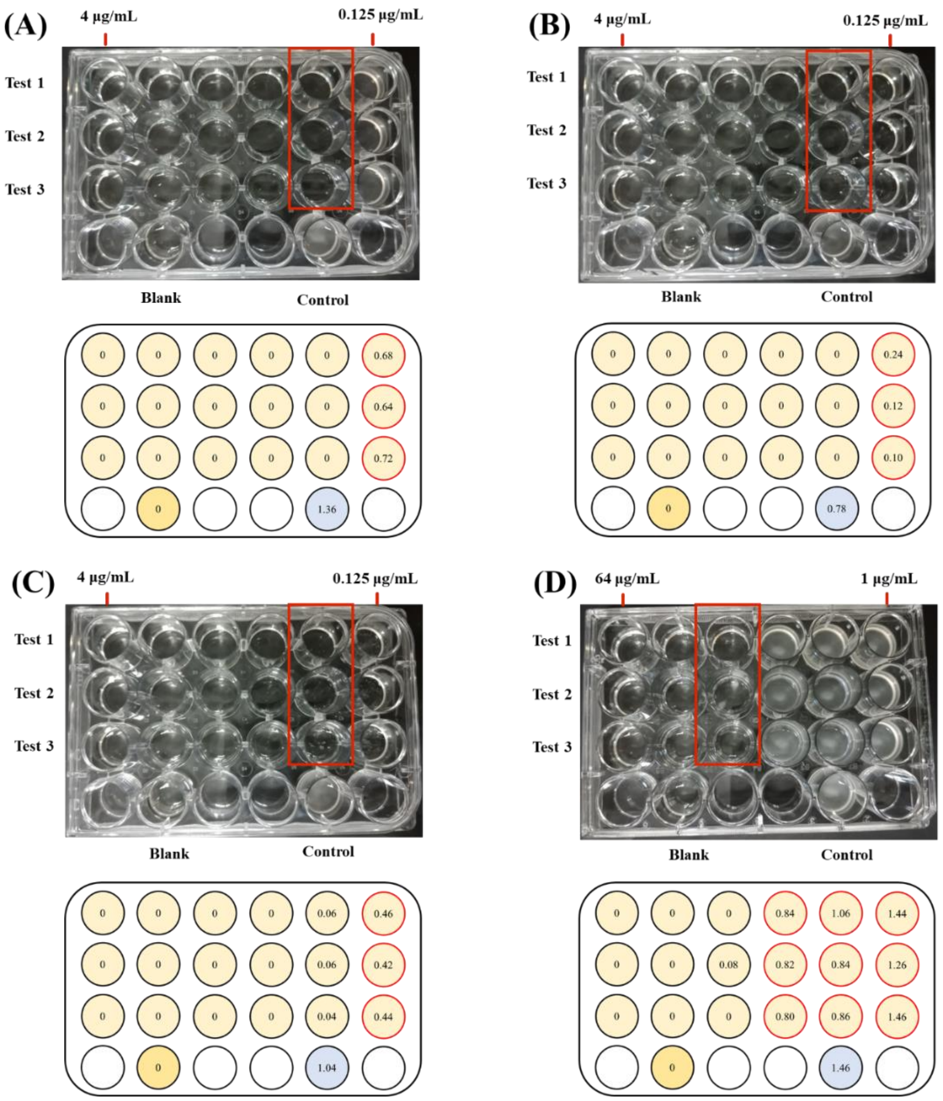

Figure S1. Corresponding photographs of the microdilution results by using oxacillin as the antibacterial agent against four model *S. aureus* strains, including (A) *S. aureus* clinical strain, (B) *S. aureus* BCRC 10823, (C) *S. aureus* BCRC 10831, and (D) MRSA. The red squares indicate where the determined MICs are. The highest and lowest concentrations were labeled on the top of the photographs to represent the 2-fold series dilution. The cartoon illustration showed the OD value obtained in each well1. Tests 1-3 indicate three replicates.

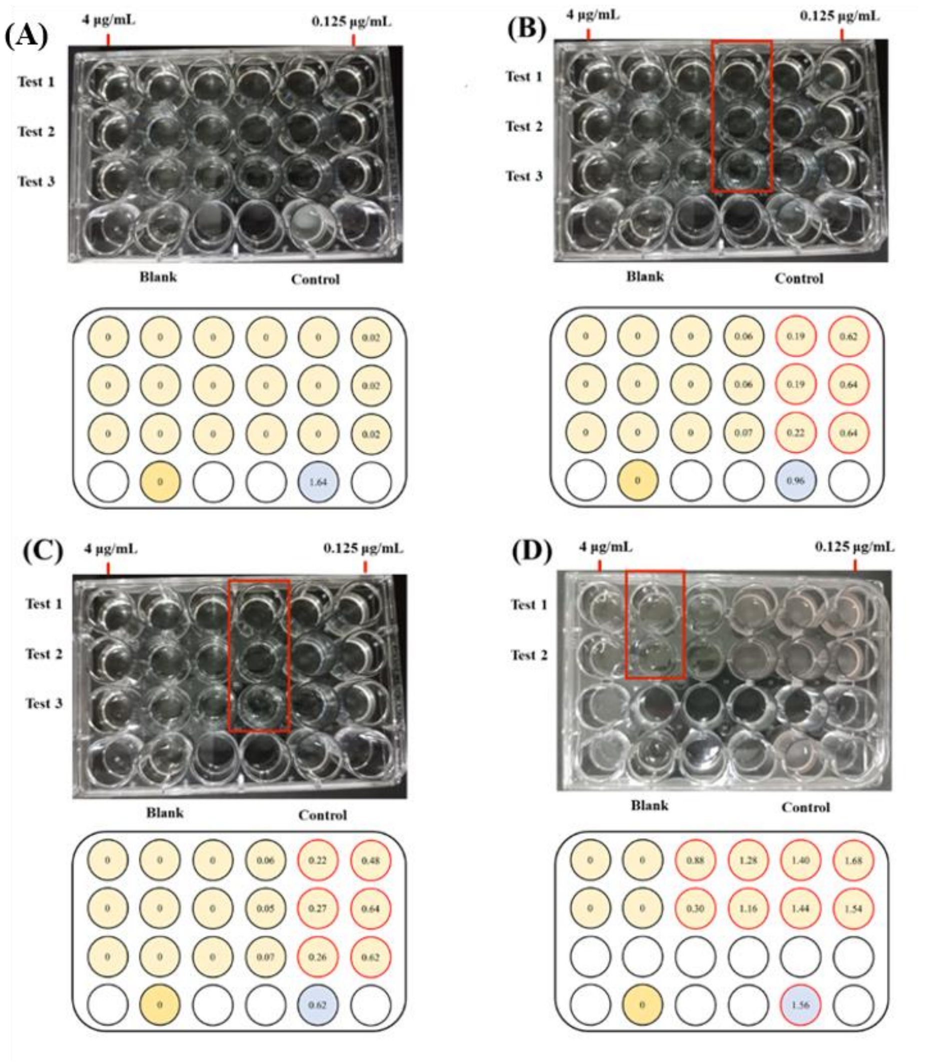

Figure S2. Corresponding photographs of the microdilution results by using vancomycin as the antibacterial agent against four model *S. aureus* strains, including (A) *S. aureus* clinical strain, (B) *S. aureus* BCRC 10823, (C) *S. aureus* BCRC 10831, (D) MRSA clinical strain. The red squares indicate where the determined MICs are. The highest and lowest concentrations were labeled on the top of the photographs to represent the 2-fold series dilution. The cartoon illustration showed the OD value obtained in each well1. Tests 1-3 indicate three replicates.

References

1. Bright, J. J.; Claydon, M. A.; Soufian, M.; Gordon, D. B., Rapid typing of bacteria using matrix-assisted laser desorption ionisation time-of-flight mass spectrometry and pattern recognition software. *J. Microbiol. Methods* **2002,** *48*, 127-138.
2. Khot, P. D.; Fisher, M. A., Novel approach for differentiating *Shigella species* and *Escherichia coli* by matrix-assisted laser desorption ionization–time of flight mass spectrometry. *J. Clin. Microbiol.* **2013,** *51*, 3711-3716.
3. Tang, W.; Ranganathan, N.; Shahrezaei, V.; Larrouy-Maumus, G., MALDI-TOF mass spectrometry on intact bacteria combined with a refined analysis framework allows accurate classification of MSSA and MRSA. *PloS one* **2019,** *14*, e0218951.
4. Wang, H.-Y.; Chung, C.-R.; Wang, Z.; Li, S.; Chu, B.-Y.; Horng, J.-T.; Lu, J.-J.; Lee, T.-Y., A large-scale investigation and identification of methicillin-resistant *Staphylococcus aureus* based on peaks binning of matrix-assisted laser desorption ionization-time of flight MS spectra. *Briefings Bioinf.* **2021,** *22*, bbaa138.
5. Kong, P.-H.; Chiang, C.-H.; Lin, T.-C.; Kuo, S.-C.; Li, C.-F.; Hsiung, C.-A.; Shiue, Y.-L.; Chiou, H.-Y.; Wu, L.-C.; Tsou, H.-H., Discrimination of methicillin-resistant *Staphylococcus aureus* by MALDI-TOF mass spectrometry with machine learning techniques in patients with *Staphylococcus aureus* bacteremia. *Pathogens.* **2022,** *11*, 586.
6. Liu, X.; Su, T.; Hsu, Y. M. S.; Yu, H.; Yang, H. S.; Jiang, L.; Zhao, Z., Rapid identification and discrimination of methicillin-resistant *Staphylococcus aureus* strains via matrix-assisted laser desorption/ionization time-of-flight mass spectrometry. *Rapid Commun. Mass Spectrom.* **2021,** *35*, e8972.
7. Ciloglu, F. U.; Caliskan, A.; Saridag, A. M.; Kilic, I. H.; Tokmakci, M.; Kahraman, M.; Aydin, O., Drug-resistant S*taphylococcus aureus* bacteria detection by combining surface-enhanced Raman spectroscopy (SERS) and deep learning techniques. *Sci. Rep.* **2021,** *11*, 18444.
8. Yu, J.; Tien, N.; Liu, Y.-C.; Cho, D.-Y.; Chen, J.-W.; Tsai, Y.-T.; Huang, Y.-C.; Chao, H.-J.; Chen, C.-J., Rapid identification of methicillin-resistant *Staphylococcus aureus* using MALDI-TOF MS and machine learning from over 20,000 clinical isolates. *Microbiol. Spectrum* **2022,** *10*, e00483-22.
